# Bilaterale orbitale Affektion bei multiplem Myelom

**DOI:** 10.1007/s00347-023-01817-5

**Published:** 2023-02-27

**Authors:** Baran Khalil, Laura Parzer, Sigrid Machherndl-Spandl, Sophie Haitchi-Petnehazy, Katharina Etmajer, Karl Haas, Peter Reinelt

**Affiliations:** 1 Augenklinik Barmherzige Brüder, Seilerstätte 2, 4020 Linz, Oberösterreich Österreich; 2 Medizinische Onkologie und Hämatologie, Elisabethinen Barmherzige Schwestern Linz, Linz, Oberösterreich Österreich; 3 Pathologie, Krankenhaus der Barmherzige Schwestern Linz, Linz, Oberösterreich Österreich; 4 Hals-, Nasen- und Ohrenheilkunde, Krankenhaus der Barmherzige Schwestern Linz, Linz, Oberösterreich Österreich

## Anamnese

Ein 55-jähriger Patient wurde mit akuten Kopfschmerzen und beidseitiger Visusminderung sowie bilateraler schmerzhafter Proptosis an der Augenabteilung vorstellig. Aufgrund einer fieberhaften Harnwegsinfektion wurde der Patient erst wenige Stunden zuvor auf der urologischen Fachabteilung stationär aufgenommen und einmalig die erste Dosis Penicillin G intravenös verabreicht. In der Anamnese gab es keine ophthalmologischen Vorerkrankungen oder Operationen. Das Sehvermögen war stets einwandfrei. Es gab keine Symptome, die auf eine Schilddrüsenerkrankung hindeuteten, wie Hitzeintoleranz, übermäßiges Schwitzen oder veränderte Stuhlgewohnheiten. Auch Symptome wie Fieber, Appetitlosigkeit oder Gewichtsverlust wurden verneint. Es bestand jedoch eine umfassende Vorgeschichte mit mehrfachen thrombotischen Ereignissen (Sinusvenenthrombose, Myokardinfarkt und Mediainsult) aufgrund einer später festgestellten heterozygoten Prothrombinmutation G20/21/0/A. Ebenso war eine neurogene Blasenentleerungsstörung nach Operation eines Tumors im Sakrum seit der Jugend bekannt. Weiterhin war 17 Tage zuvor eine Immunisierung gegen COVID-19 erfolgt.

## Befund

**Figure 1:**
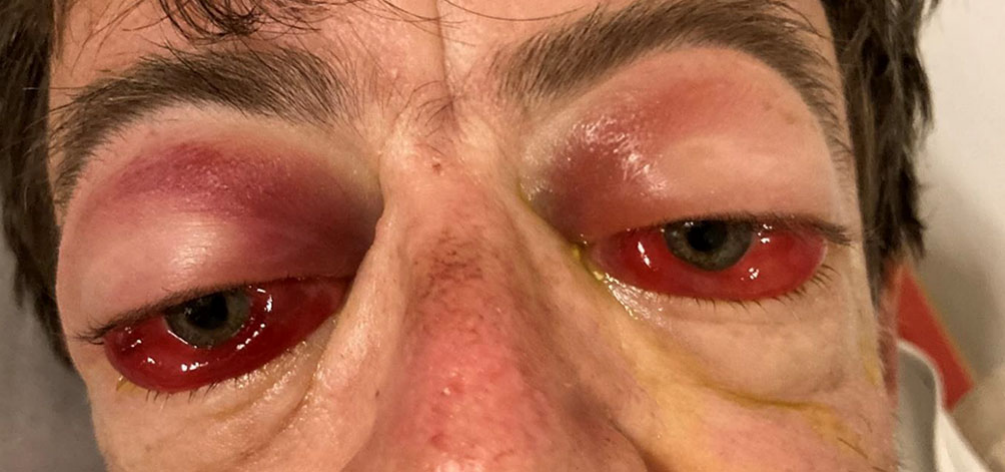

Bei der notfallmäßigen Vorstellung betrug der bestkorrigierte Visus Handbewegung am rechten Auge und Lichtwahrnehmung am linken Auge. Beidseits bestand eine totale Ophthalmoplegie ohne Lichtreaktion der Pupille. Der intraokulare Druck lag beidseitig bei 20 mm Hg. Es bestanden eine ausgeprägte bilaterale Proptosis und Chemosis der Bindehaut (Abb. 1). Auf eine Exophthalmometermessung nach Hertel wurde aufgrund des akuten Geschehens verzichtet. Die Vorderkammer war unauffällig und in der Ophthalmoskopie konnte eine Papillenschwellung ausgeschlossen werden. Insbesondere fanden wir keine Anzeichen für Gefäßprobleme in beiden Augen und es gab keine Netzhaut- oder Aderhautblutungen.

Im Labor zeigten sich eine erhöhte Erythrozytensedimentationsrate (Blutkörperchensenkungsgeschwindigkeit) von 33 mm/h und ein Anstieg des CRP auf 21 mg/dl. Die Schilddrüsenparameter lagen im Normbereich (einschließlich TSH, fT_4_/fT_3_, Anti-TPO, Anti-TRAK). Wir fanden keine IgG4-assoziierte Erkrankung, da die anfänglichen und nachfolgenden Blutuntersuchungen normale Werte ergaben.

**Figure 2:**
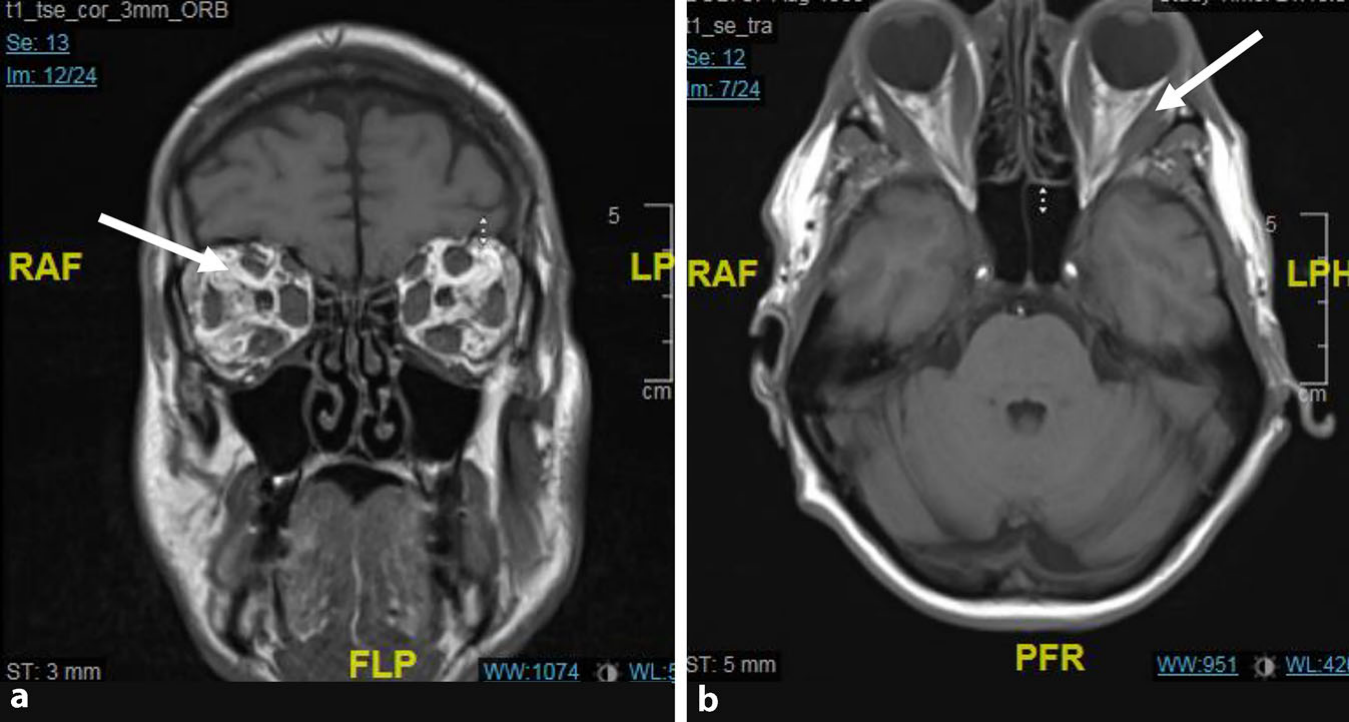

Bildgebend (Abb. 2) zeigte sich eine massive Schwellung der gesamten Augenmuskulatur einschließlich des retrobulbären Fettgewebes. Es kam zu einer Fettherniation durch die Fissura orbitalis superior, sowie zu einer Dehnung beider Sehnerven.

## Therapie und Verlauf

Neben einer i.v.-Hochdosistherapie mit Prednisolon, verabreichten wir systemisch Acetazolamid und eine Schmerzmedikation. Außerdem wurde die systemische antibiotische Therapie umgestellt. Am nächsten Morgen zeigte der Patient eine deutliche Befundverbesserung. Die Pupillenreaktion normalisierte sich und es gab keine Anzeichen einer Ophthalmoplegie. Es zeigte sich eine freie Bulbusmotilität, und der bestkorrigierte Visus lag nach einem Tag bei 0,6 für beide Augen und nach 2 Tagen bei 1,0 für beide Augen.

Aufgrund des raschen Ansprechens auf die i.v.-Steroidtherapie beschlossen wir, diese für 2 weitere Tage zu wiederholen und behielten danach eine Dosis von 1 mg/kgKG für 5 Wochen bei.

Im Rahmen der Durchuntersuchung fanden wir bei der Immunfixationselektrophorese im Serum aus peripherem Blut monoklonale λ‑Leichtketten, was auf eine klonale Plasmazellerkrankung hindeutet. Leichtketten sind inkomplette Immunglobuline, die aus leichten Eiweißketten bestehen. Beim multiplen Myelom kommt es zu unkontrolliertem Wachstum von Plasmazellen, vorwiegend im Knochenmark, welche Immunglobuline bilden. Das Leichtkettenmyelom ist der dritthäufigste Subtyp des multiplen Myeloms mit ca. 20–25 % der Fälle. Der Patient wurde in weiterer Folge zur Diagnosesicherung mittels Knochenmarkbiospie und evtl. Initialisierung einer Therapie einer hämatoonkologischen Abteilung zugewiesen.

Die Knochenmarkbiopsie ergab den Nachweis von ungefähr 15 % λ‑klonalen Plasmazellen. Im nächsten Schritt wurde ein Staging des Patienten durchgeführt. Es folgten eine Ganzkörper Computertomographie sowie ein PET-CT. Hierbei zeigten sich fragliche Osteolysen im Bereich S1 sowie im Bereich der Femurhälse beidseits. Üblicherweise wäre eine MRT-Bildgebung zur Abgrenzung eines „Smouldering“-Myeloms vom behandlungsbedürftigen Myelom sinnvoll, welches jedoch aufgrund mehrerer Metallclips im Os sacrum kontraindiziert war. Aufgrund des Plasmazellanteils >10 % und der mutmaßlichen Osteolysen wurde die Diagnose eines multiplen Myeloms gestellt.

Es erfolgten die Vorstellung im onkologischen Tumorboard und die Empfehlung zur Erstlinientherapie nach der Leitlinie der Deutschen Gesellschaft für Hämatologie und Medizinische Onkologie [14] mit Bortezomib, Lenalidomid sowie Dexamethason. Zusätzlich wurde Daratumumab, ein CD-38-Antikörper, gemäß den lokalen Therapieleitlinien des Tumorzentrums verabreicht. Die Initialisierung dieser Therapie bedarf zunächst der Bewertung anhand sog. SLiM-CRAB-Kriterien der International Myeloma Working Group [1]. Die Erfüllung eines Kriteriums ist hierbei ausreichend und in unserem Fall lagen ein freier Leichtkettenquotient im Serum >100 mg/l sowie suspekte Osteolysen vor.

**Figure 3:**
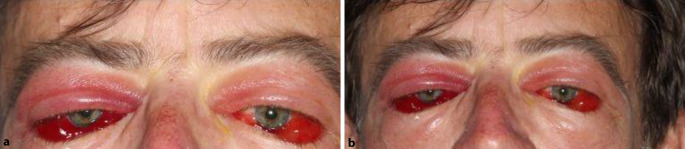

**Figure 4:**
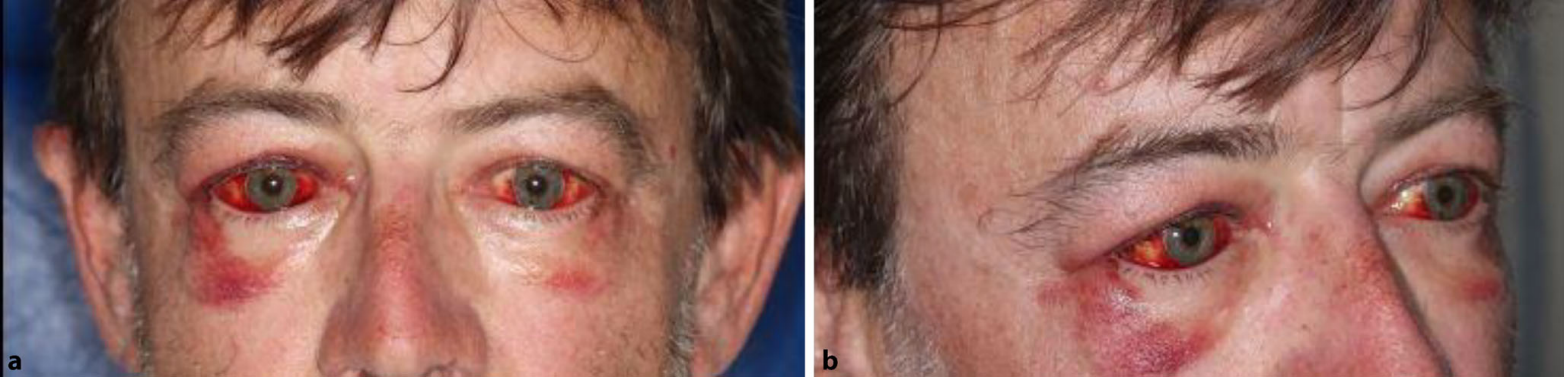

Im Rahmen der onkologischen Therapie wurde der Patient immer wieder an der Augenabteilung vorstellig. Insbesondere bei erneuten Schüben mit Augenbeteiligung in den nachfolgenden Monaten wurde immer wieder Steroid i.v. verabreicht, da dies immer zur Befundbesserung führte und die orbitale Proptosis bereits am nächsten Tag rückläufig war (Abb. 3 und 4). In zeitlichem Zusammenhang dieser Schübe wurden Harnwegsinfekte, COVID-19-Teilimpfungen und die onkologische Therapie beobachtet. So kam es beispielsweise unter der Gabe der immunmodulatorischen Substanz Lenalidomid zu einer erneuten Proptosis. Diese zeigte sich ebenfalls unter Steroidgabe schnell rückläufig.

**Figure 5:**
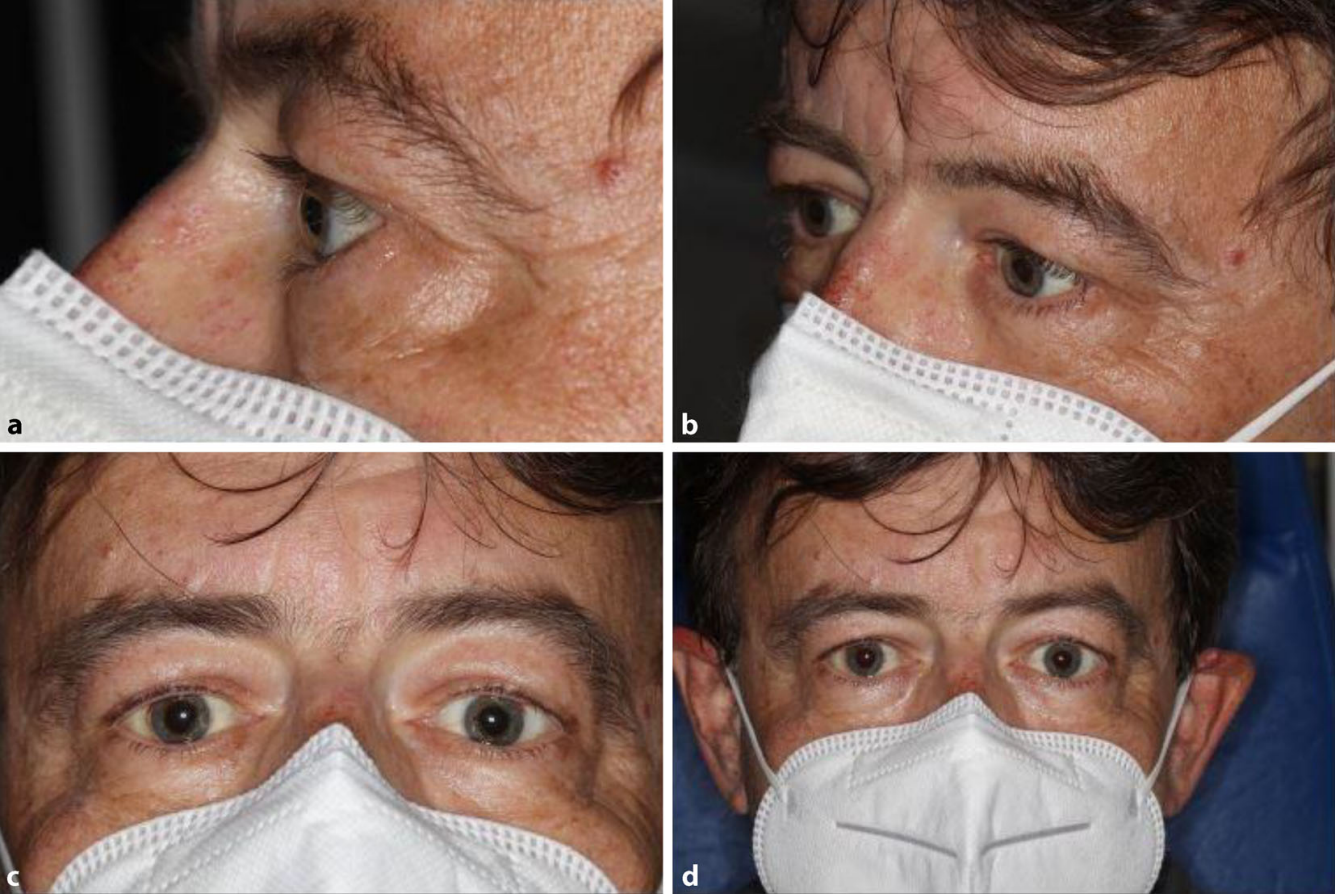

**Figure 6:**
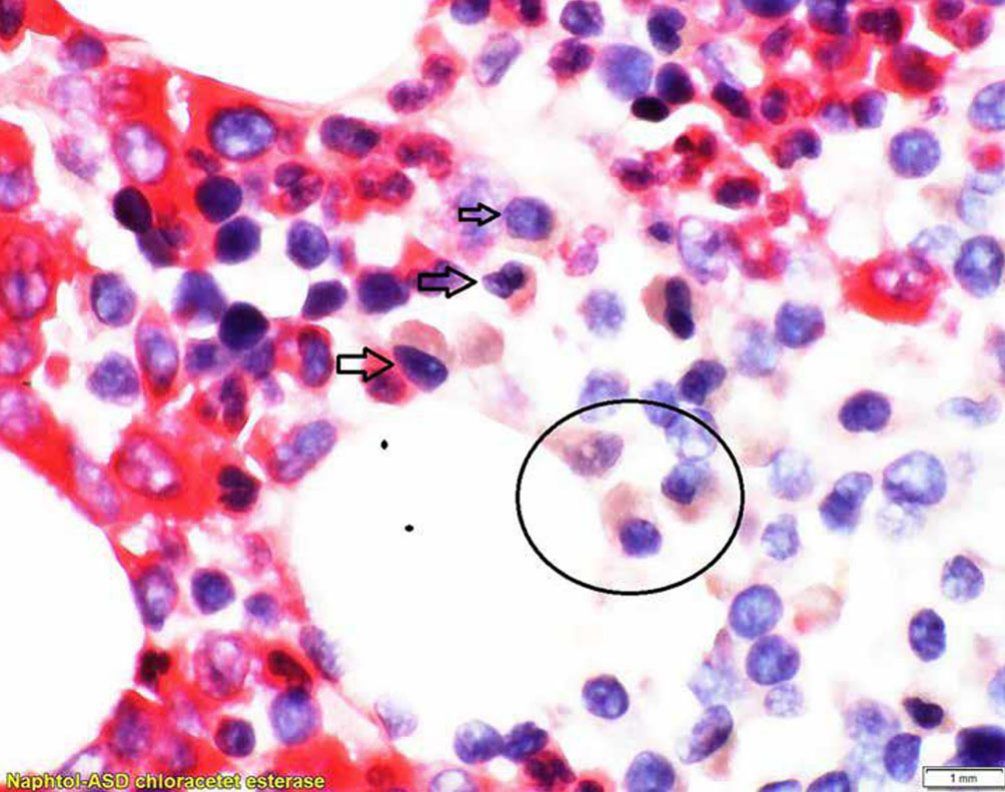

**Figure 7:**
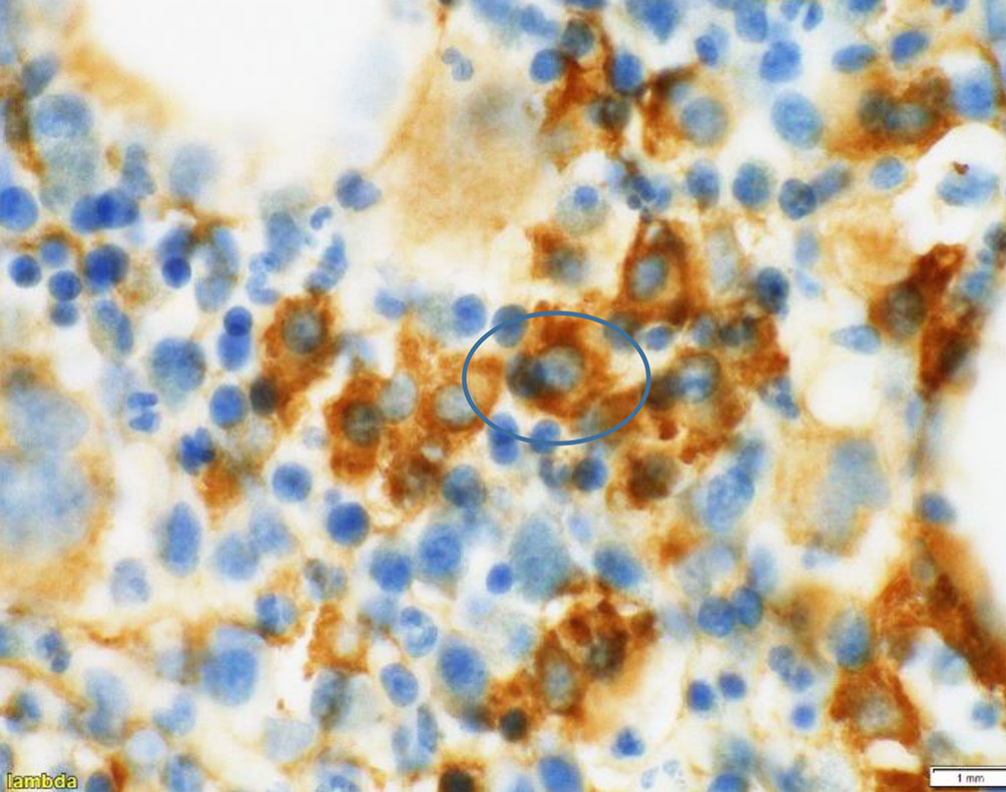

## Diagnose

Multiples Myelom

## Diskussion

Es gibt 4 verschiedene Typen einer Plasmazellneoplasie. Darunter fallen das multiple Myelom, die „monoclonal gammopathy of undetermined significance“ (MGUS), die Amyloidose und das Plasmozytom [3]. Das multiple Myelom mit orbitaler Beteiligung ist selten. Frühere Arbeiten bestätigen, dass die orbitale Präsentation vornehmlich als Proptosis in Erscheinung treten kann [4–7]. Hierbei geht die erstmalige Beschreibung auf Rodman 1972 und Chohan 1971 zurück [4]. Typischerweise geht der extramedulläre Befall mit Schmerzen und Knochenerosion einher. Außerdem finden sich häufiger solitäre Plasmozytomabsiedelungen in Form von kompakter orbitaler Tumormasse [8]. In sehr seltenen Fällen findet sich ein multiples Myelom jedoch ohne Knochenerosion oder solide Tumormasse, sondern in Form von Plasmazellablagerungen im orbitalen Gewebe [9, 10]. In unserem Fall konnten weder orbitale Knochenläsionen noch solitäre Tumorlokalisationen gefunden werden. Klinischer Leitbefund bei unserem Patienten waren lediglich die Schwellung des orbitalen Fett- und Muskelgewebes.

Analog zur endokrinen Orbitopathie entschieden wir uns in der Akuttherapie für die i.v.-Gabe einer Megadosis mit Steroiden, um eine rasche Abschwellung des Gewebes zu erreichen. Eine gute Steroidresponsibilität ist bei der endokrinen Orbitopathie bzw. der idiopathischen entzündlichen Orbitaerkrankung bereits beschrieben [11]. Zum Zeitpunkt der ersten Gabe war die Grunderkrankung noch nicht bekannt. Die Behandlung einer bilateralen orbitalen Proptosis unklarer Ursache ist daher eine Einzelfallentscheidung. Es wurden bei unserem Patienten in der Kurzzeitbehandlung mit Hochdosissteroiden keine adversen Nebenwirkungen festgestellt. Der Wirkmechanismus liegt einerseits in der Inhibition der Phospholipase A2 und der Zyklooxygenase sowie andererseits in einem immunsuppressiven Effekt [12]. In den wenigen beschriebenen Fallberichten wird von spontanen Rückbildungen [7], einer Kombinationstherapie von Steroiden und Chemotherapie [3, 13] sowie gar einem akuten operativen Vorgehen mittels lateraler Kanthotomie und Kantholyse berichtet [6].

In der Langzeitbehandlung ist die Erstlinientherapie des multiplen Myeloms mit einer Kombinationstherapie aus Chemo‑/Immuntherapeutika der Goldstandard, um das Überleben zu verlängern. Im Anschluss daran wird bei geeigneten Patienten (keine schweren Komorbiditäten, biologisches Alter <70 Jahre) eine autologe Stammzelltransplantation empfohlen, mit nachfolgender Erhaltungstherapie. In unserem Fall wurde die Kombination Bortezomib, Lenalidomid und Dexamethason gewählt, deren Wirksamkeit in Phase-III-Studien bereits bestätigt wurde. Der Zeitpunkt der Stammzellsammlung wird primär oder in Abhängigkeit vom Ansprechen festgelegt. Häufig erfolgt die Stammzellsammlung nach 4 Zyklen. Die 5‑Jahres-Überlebensrate bei unserem Patienten im Stadium ISS I und R‑ISS I nach dem „International Staging System“ beträgt 82 % [14].

Auch nach Erreichen einer kompletten Remission ist bei der großen Mehrheit der Patienten mit molekulargenetischen Methoden, Durchflusszytometrie, MRT oder PET, eine minimale Resterkrankung nachzuweisen [2].

Differenzialdiagnostisch mussten mehrere Erkrankungen ausgeschlossen werden. Dazu gehören eine Sinusvenenthrombose, eine orbitale Zellulitis sowie auch orbitale oder zerebrale Raumforderungen. Pathologische Parameter, wie erhöhte Blutkörperchensenkung, CRP-Erhöhung sowie LDH-Erhöhung sind im Zusammenhang mit dem multiplen Myelom bereits beschrieben [13]. Letztlich führten klonale λ‑Leichtketten zur Diagnose des multiplen Myeloms. Die Knochenmarkbiopsie bestätigte letztendlich die Diagnose.

Weitere Merkmale eines multiplen Myeloms können direkte Infiltrate, Kompression oder Verdrängung von Gewebe und Nerven oder ein Hyperviskositätssyndrom umfassen. Dies wiederum kann zu Hyperviskositätsretinopathie, Diplopie oder Ablagerungen von Leichtketten im Augengewebe führen [9].

Da die Diagnose aus der Knochenmarkbiopsie bereits gesichert war, lehnte der Patient eine weitere orbitale Biopsie ab. Entscheidend für den Patienten sind dennoch eine rasche Diagnosestellung sowie ein frühzeitiger Therapiebeginn.

## Fazit für die Praxis

- Das multiple Myelom ist eine klonale Knochenmarkerkrankung mit sehr heterogenem klinischem Erscheinungsbild, das u. a. hämatopoetische Insuffizienz, renale Funktionseinschränkung und/oder ausgeprägte Osteodestruktion umfassen kann und in seltenen Fällen auch zu einem extramedullären Befall führen kann. Die orbitale Beteiligung ist selten.
- Bilaterale Proptosis und Chemosis aufgrund einer Schwellung der orbitalen Adnexe sind als paraneoplastische Erscheinungsbilder beschrieben. Im Extremfall kann dies zu einer Kompression des Sehnervs führen.
- Typischerweise ist eine rasche Rückbildung der orbitalen Proptosis durch i.v.-Gabe von Kortikosteroiden als klinischer Hinweis auf ein Ansprechen z. B. einer Lymphom- oder Myelomerkrankung auf immunsuppressive Therapie zu werten. Maßgeblich für die Diagnose einer malignen Plasmazellerkrankung ist jedoch der Nachweis eines Paraproteins oder pathologischer freier Leichtketten im peripheren Blut.
- Die Diagnosesicherung des Myeloms erfolgt durch eine Knochenmarkbiopsie.
- Die langfristige Therapie liegt in einer systemischen Chemoimmuntherapie, kombiniert mit einer autologen Stammzelltransplantation.
- Der Augenarzt kann durch ein schnelles Eingreifen einen Visusverlust durch Optikuskompression verhindern und zur raschen Diagnosefindung wesentlich beitragen, um damit das Überleben des Patienten verlängern zu können.
