# Erratum zu: Bilaterale orbitale Affektion bei multiplem Myelom

**DOI:** 10.1007/s00347-023-01851-3

**Published:** 2023-04-19

**Authors:** Baran Khalil, Laura Parzer, Sigrid Machherndl-Spandl, Sophie Haitchi-Petnehazy, Katharina Etmajer, Karl Haas, Peter Reinelt

**Affiliations:** 1 Augenklinik Barmherzige Brüder, Seilerstätte 2, 4020 Linz, Oberösterreich Österreich; 2 Medizinische Onkologie und Hämatologie, Elisabethinen Barmherzige Schwestern Linz, Linz, Oberösterreich Österreich; 3 Pathologie, Krankenhaus der Barmherzige Schwestern Linz, Linz, Oberösterreich Österreich; 4 Hals-, Nasen- und Ohrenheilkunde, Krankenhaus der Barmherzige Schwestern Linz, Linz, Oberösterreich Österreich

**Erratum zu:**

**Ophthalmologie 2023**

10.1007/s00347-023-01817-5

In der Legende zu **Abb. 1** hat sich versehentlich ein Fehler eingeschlichen: Die Legende lautet korrekt:

**Abb. 1** Erste Präsentation mit akuter bilateraler Proptosis und Chemosis der Bindehaut, totale Ophthalmoplegie und fehlender Pupillenreaktion

Wir bitten Sie, die korrigierte Abbildungslegende zu beachten und den Fehler zu entschuldigen.

Der vollständige und korrigierten Artikel steht Ihnen auf www.springermedizin.de zur Verfügung. Bitte geben Sie dort den Beitragstitel in die Suche ein.

